# Anterior sling with hamstring autograft for recalcitrant reverse shoulder arthroplasty instability

**DOI:** 10.1097/RC9.0000000000000164

**Published:** 2026-02-05

**Authors:** Pedro Amaro, Luís Pires

**Affiliations:** Hospital Beatriz Ângelo, Lisboa, Portugal & Hospital da Luz, Torres de Lisboa, Portugal

**Keywords:** complications, proximal humerus fracture, reverse total shoulder arthroplasty, shoulder arthroplasty dislocation, shoulder replacement instability

## Abstract

**Introduction and importance:**

Reverse total shoulder arthroplasty (RTSA) has significantly advanced the treatment of a wide range of shoulder disorders, expanding its indications from rotator cuff arthropathy to include irreparable rotator cuff tears, complex fractures, and inflammatory arthritis. However, the rapid increase in RTSA procedures has been accompanied by a corresponding rise in complications, with dislocation being one of the most prevalent early postoperative complications.

**Case presentation:**

This case report presents a 74-year-old, right-hand dominant female patient with a periprosthetic fracture of the right shoulder. She underwent revision surgery involving glenoid lateralization and the use of a thicker, restrictive polyethylene insert. Postoperatively, the patient experienced pain and instability, eventually leading to an atraumatic anterior dislocation. A re-revision surgery was performed, incorporating an anterior sling using a hamstring autograft to address subscapularis deficiency caused by recurrent dislocations, and an increased humeral tray size for improved containment. Approximately 2 years after the re-revision surgery, the patient demonstrated restored stability, improved range of motion, and reported satisfaction with the outcome.

**Clinical discussion:**

Numerous recommendations for addressing instability or dislocation following RTSA have been discussed in the literature. Dislocation may be influenced by several biomechanical factors, including humeral shortening, excessive medialization, implant version, socket constraint, soft-tissue tensioning, and nerve dysfunction. Subscapularis deficiency is a potential contributor to recurrent dislocations. Few techniques have been described to manage and enhance soft tissue tension in cases of subscapularis insufficiency.

**Conclusion:**

This case report highlights the challenges of managing recurrent anterior dislocation following RTSA. It demonstrates the successful use of an anterior hamstring autograft sling as a salvage procedure to restore stability in cases of persistent anterior dislocation after RTSA. However, further clinical studies are needed to better define the role of such interventions in addressing complications associated with RTSA.

## Introduction

Reverse total shoulder arthroplasty (RTSA) has demonstrated good to excellent clinical outcomes, leading to a significant increase in its use over recent years. However, this rise in RTSA procedures has been accompanied by a proportional increase in associated complications^[^1^]^.

HIGHLIGHTS

- Managing dislocation or instability after revision reverse total shoulder arthroplasty (RTSA) remains a significant challenge.
- In the setting of RTSA, subscapularis deficiency can increase the risk of instability, and the necessity of repairing the subscapularis during RTSA remains a topic of ongoing debate.
- We describe a soft tissue reconstruction technique with a hamstring autograft for the management of instability in RTSA, in cases of subscapularis deficiency.

Among these, instability following RTSA presents as a complex, multifactorial problem that, if not adequately addressed, can result in a nonfunctional upper extremity. Contributing factors to instability include inadequate arm length restoration, insufficient soft tissue tension, component malposition, bony impingement, polyethylene liner wear, infection, and axillary nerve dysfunction^[^2,3^]^.

Managing dislocation or instability after revision RTSA remains a significant challenge. While adjusting component positions and restoring arm length with appropriate soft tissue tension can resolve many cases of instability, some patients may experience recurrent dislocations despite these interventions. In this case report, we present the successful management of recurrent anterior dislocation following RTSA, utilizing an anterior sling with hamstring autograft.

To our knowledge, this is the first case report documenting the use of soft tissue reconstruction with a hamstring autograft for the management of instability in RTSA.

## Case report

**Figure 1.**
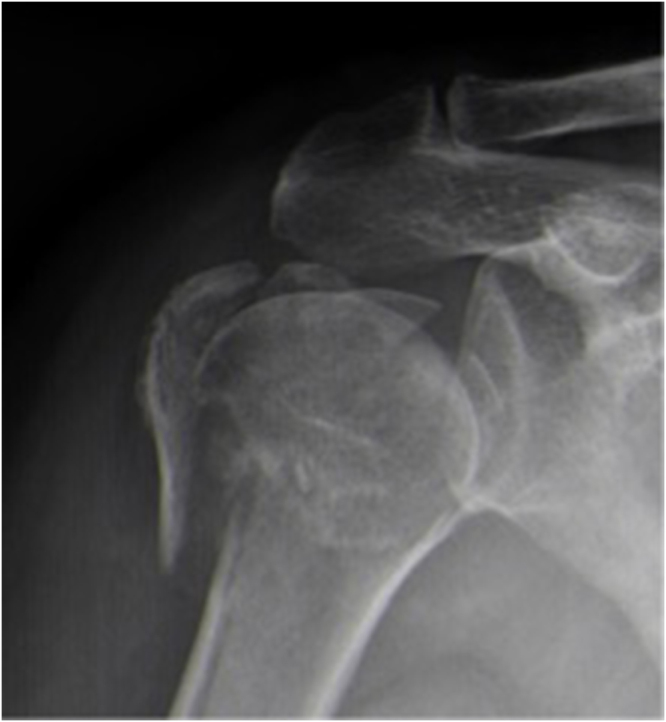
Right shoulder Neer IV proximal humerus fracture.

**Figure 2.**
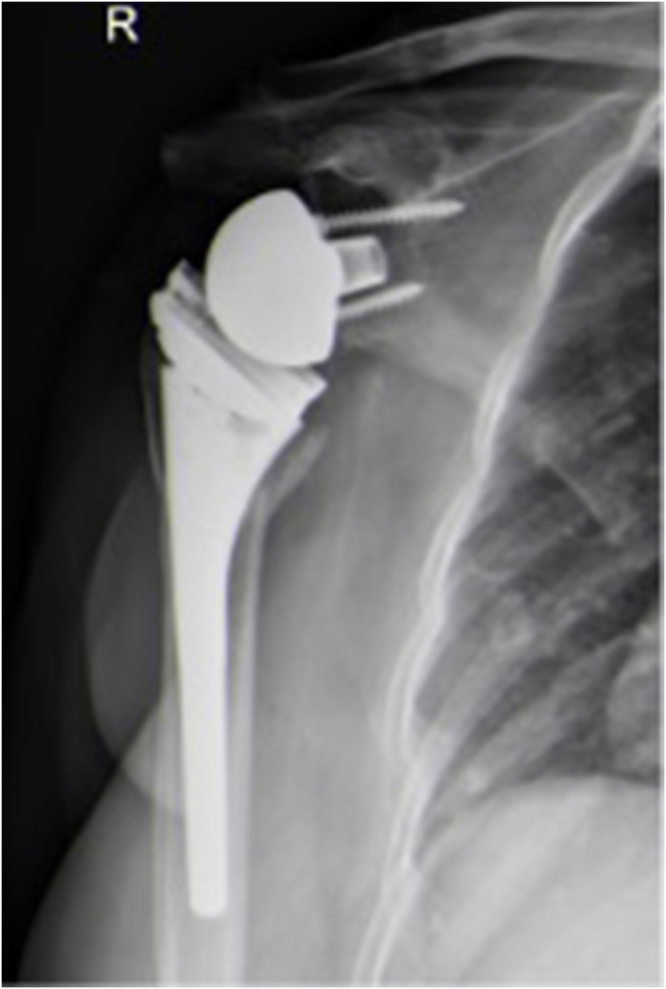
Postoperative X-ray of primary reverse shoulder arthroplasty.

The patient is a 74-year-old female who sustained a fall three years ago, resulting in a Neer 4-part proximal humerus fracture of the right shoulder (Fig. 1). She underwent RTSA with excellent postoperative range of motion, good shoulder function, and satisfaction with the outcome (Fig. 2).

**Figure 3.**
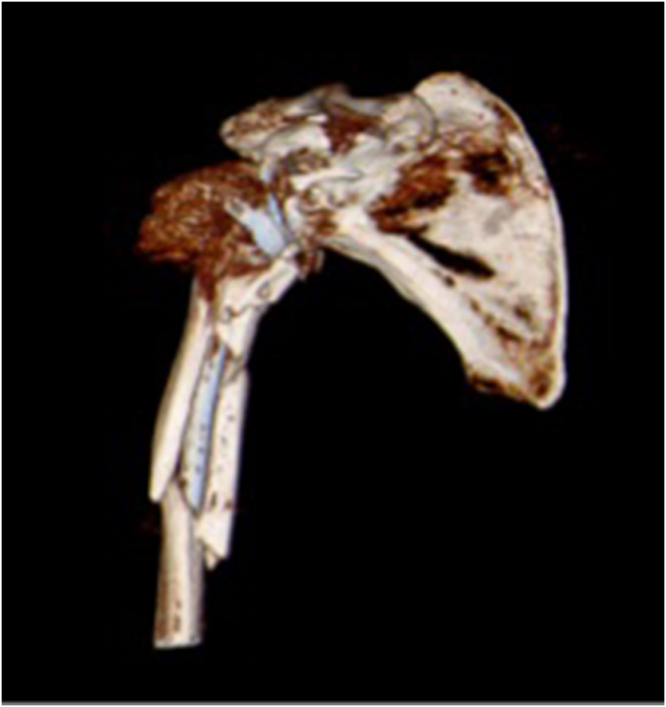
Wright and Cofield Type B comminuted periprosthetic fracture.

**Figure 4.**
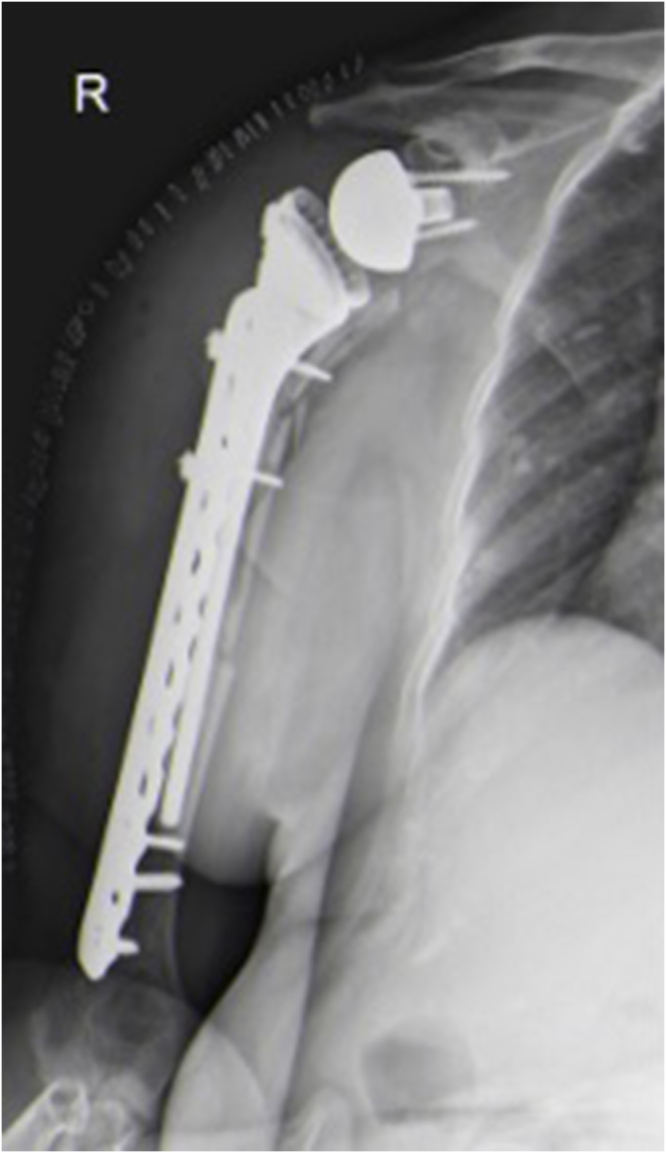
Postoperative X-ray of open reduction internal fixation and long-stem revision of periprosthetic fracture.

One year later, the patient fell again and was diagnosed with a right shoulder Type B periprosthetic fracture according to the Wright and Cofield classification, as well as a greater tuberosity fracture dislocation of the left shoulder (Fig. 3). The left shoulder fracture was managed conservatively with closed reduction, while the right periprosthetic fracture was treated with open reduction, internal fixation, and long-stem revision (Fig. 4).

Two weeks after the fracture, the patient sustained another fall and presented to the emergency department with a right RTSA dislocation and a left shoulder dislocation. Full-length bilateral calibrated arm radiographs revealed no humeral shortening. Intraoperative cultures were negative for infection. The patient underwent revision surgery with glenoid lateralization (36 + 4 mm) and a +9 mm constrained polyethylene insert on the humeral side.

**Figure 5.**
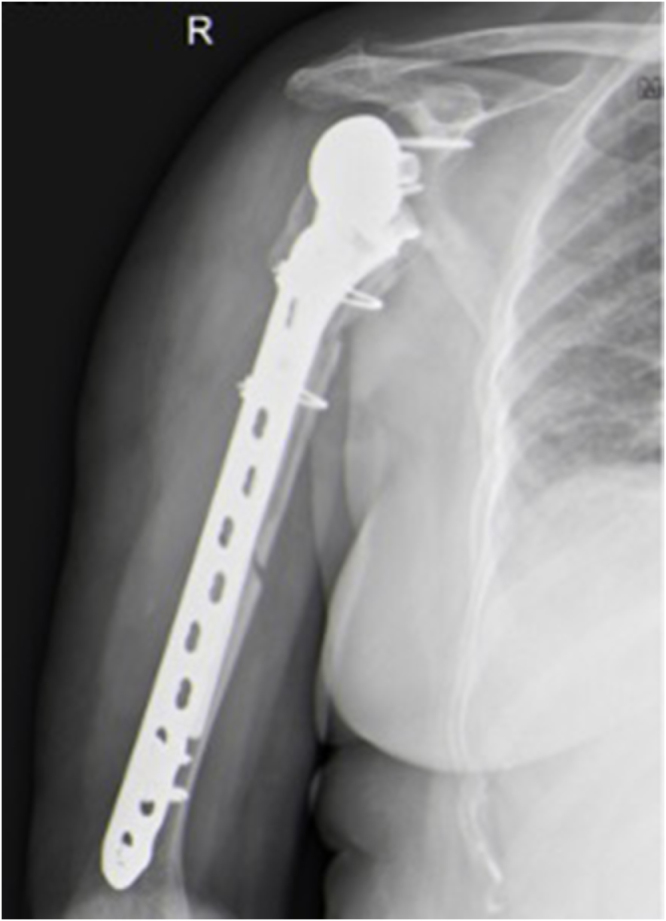
X-ray of second dislocation after first revision surgery.

Two weeks after the revision surgery, the patient experienced another dislocation (Fig. 5). A CT scan and X-rays revealed that both the glenoid and humeral implant versions, as well as glenoid tilt, were normal. There were no signs of bony or soft tissue impingement. Additionally, a thorough physical examination and electromyography showed no nerve dysfunction, including the axillary nerve. Joint aspiration and laboratory tests were negative for infection. Given the patient’s pain and inability to use her arm, a discussion was held regarding her treatment goals. Following this, she opted to proceed with a revision RTSA, utilizing an anterior sling with a hamstring autograft.

The patient was placed in the beach chair position, and the shoulder was approached through the previous deltopectoral incision. The shoulder was reduced, and external and internal rotation of the humerus showed no evidence of impingement. Dislocation occurred with the arm in extension and internal rotation. Intraoperatively, no implant loosening was observed, but a subscapularis tear was noted. The subscapularis was medially retracted, with poor tendon quality, and was irreparable. The humeral tray and glenosphere were removed, and a hamstring autograft harvest was performed using the standard technique for harvesting the semitendinosus and gracilis tendons.

**Figure 6.**
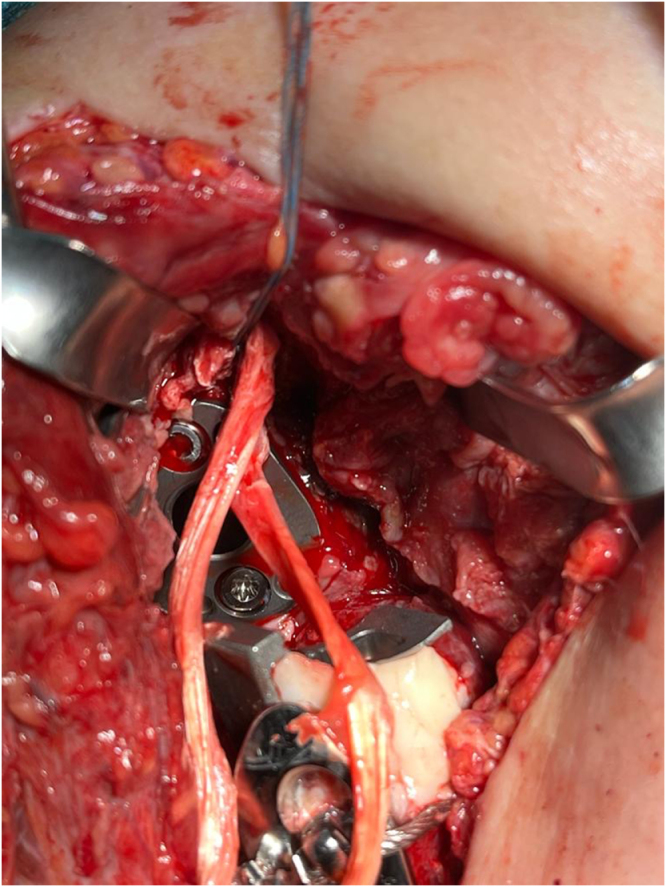
Suture anchor insertion on the anterior glenoid.

**Figure 7.**
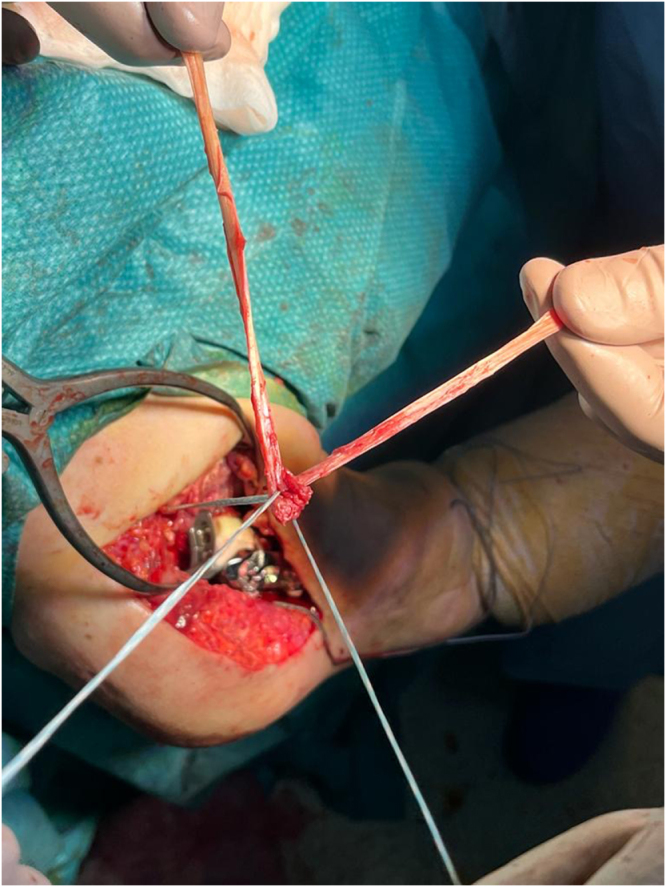
Hamstring autograft preparation using the Krakow suture technique.

**Figure 8.**
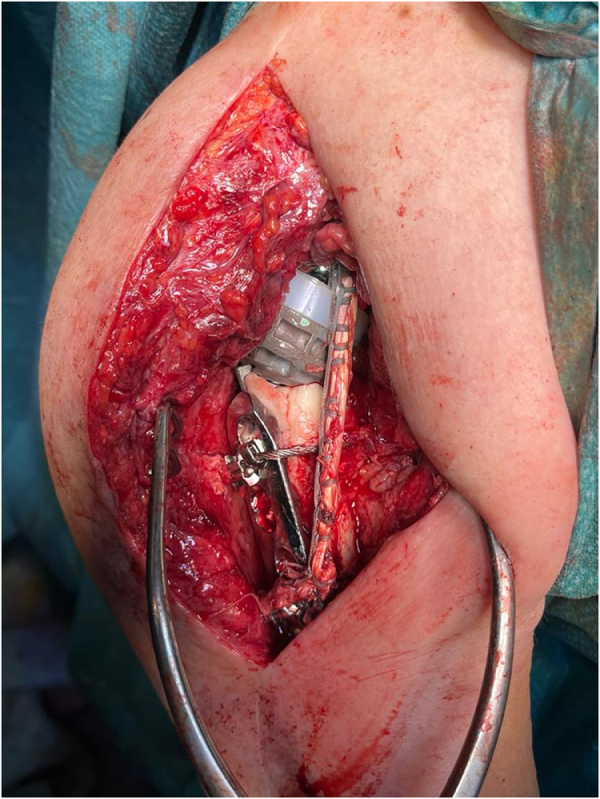
Intraoperative picture of the final construct.

A 4.5 mm suture anchor loaded with two tapes was placed anteriorly, medial to the glenoid face, and lateral to the coracoid base (Fig. 6). A Krakow suture technique was used to prepare the hamstring autograft (Fig. 7). The autograft was fixed 5–7 cm distally through transosseous bone tunnels on the medial side of the proximal humerus (Fig. 8). The same glenoid lateralization (36 + 4 mm) and +9 mm constrained polyethylene insert were used on the humerus. Intraoperative stability assessment was performed, and the shoulder was moved through an arc of motion to ensure 90° of forward flexion, 45° of external rotation, and sufficient internal rotation, confirming that a functional range of motion could be achieved without significant graft tension. See Supplemental Digital Content Video 1, available at: http://links.lww.com/IJSCR/A7, for intraoperative stability testing. The incision was then closed using standard techniques.

**Figure 9.**
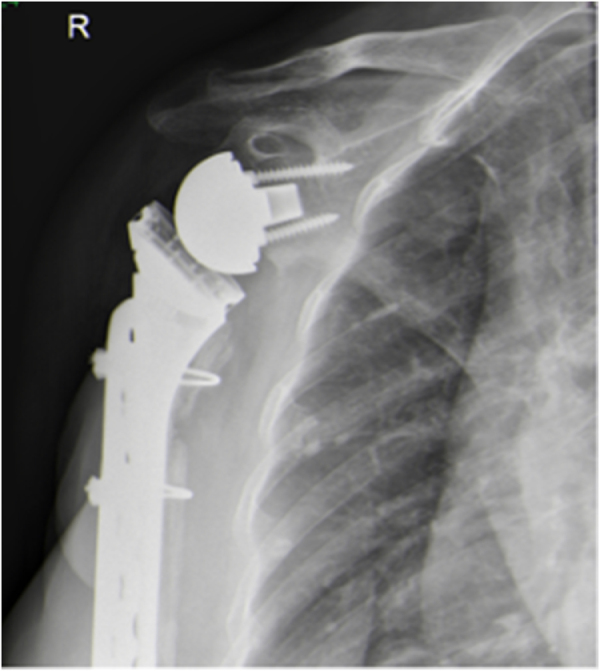
Re-revision postoperative X-ray.

Postoperatively, the patient was placed in a sling for 3 weeks. Passive range of motion was allowed until week six, after which progressive range of motion was initiated under the supervision of physical therapy (Fig. 9).

At the final evaluation, 2 years postoperatively, the patient had a stable shoulder joint with active forward flexion of 80°, external rotation of 10°, and internal rotation to the sacroiliac joint. Passive range of motion revealed 140° of forward flexion, 50° of external rotation, and internal rotation to the 5th lumbar vertebra. The patient reported a pain score of 1/10 on the visual analog scale, was able to perform limited activities of daily living, with a 57-point ASES score, and expressed overall satisfaction with the procedure. This work has been reported in line with the SCARE criteria^[^4^]^.

## Discussion

Several treatment options exist for managing persistent instability following RTSA. The most commonly employed strategies to restore stability include humeral length restoration and glenoid lateralization. Humeral length can be restored using augmentations for minor deficiencies or through stem revision, with or without proximal humeral augmentation, in cases of more significant bone loss^[^5^]^.

Glenoid lateralization can be achieved by utilizing a larger glenosphere, an augmented lateralized glenosphere, or through bony lateralization of the glenoid using a graft. Additionally, constrained liners may enhance stability, and repair of the subscapularis tendon can further contribute to joint stability^[^5,6^]^.

RTSA has become a common treatment for rotator cuff arthropathy, and its indications have expanded to include a range of conditions such as proximal humerus fractures, irreparable rotator cuff tears, tumors, arthritis, and more^[^7^]^.

However, like any surgical procedure involving prosthetic implantation, RTSA carries a risk of various complications. These may include prosthetic instability, scapular notching, acromial fractures, infection, periprosthetic fractures, deltoid weakness, and implant loosening^[^8–10^]^.

One of the most common complications in the early postoperative period is dislocation, with rates ranging from 1.5% to 31%^[^11,12^]^.

Numerous recommendations for addressing instability or dislocation following RTSA have been discussed in the literature. Key considerations include identifying the mechanism and underlying pathology of the instability, selecting appropriate implant sizes, evaluating the potential use of various prosthetic designs, and ensuring surgical expertise to manage scar tissue near neurovascular structures. Additionally, surgeons must address factors such as arm length restoration, bony impingement, component malposition, component wear, and soft-tissue laxity. Despite these considerations, managing unstable RTSA remains a challenging task, with reported failure rates for revision RTSA ranging from 15% to 40%. Dislocation may be influenced by several biomechanical factors, including humeral shortening, excessive medialization, implant version, socket constraint, soft-tissue tensioning, and nerve dysfunction^[^2,11,13^]^.

In the first revision surgery, our primary objective was to enhance soft tissue tension, as both the glenoid and humeral implant versions, along with glenoid tilt, appeared normal on computed tomography scan (CT scan) and X-ray imaging. Additionally, a comprehensive physical examination and electromyography revealed no evidence of nerve dysfunction, including the axillary nerve. Despite these efforts, anterior dislocation recurred after the revision surgery, prompting us to consider subscapularis deficiency as a potential contributor to the recurrent dislocations.

During the final revision surgery, we implemented an anterior sling using a hamstring autograft. As a result, the patient has not experienced any further dislocations even 2 years postoperatively, and she reported a complete absence of instability.

In the context of RTSA, subscapularis deficiency can lead to significant complications. However, the necessity of repairing the subscapularis during RTSA remains a topic of ongoing debate. Some studies suggest that whether the subscapularis is repaired, complication and dislocation rates do not significantly differ^[^14–16^]^.

Edwards *et al*. reported an increased risk of instability when the subscapularis tendon was not repaired. Furthermore, Collin *et al*. advocated for the repair of reparable subscapularis tendons during RTSA whenever possible. However, there are instances where the subscapularis is deemed irreparable, either during the initial RTSA or, as in our case, during revision surgery. Given that subscapularis deficiency can lead to complications, alternative treatment strategies may be required^[^17,18^]^.

Kany *et al*^[^19^]^ suggested using RTSA in conjunction with anterior latissimus dorsi transfer, while Baek *et al*^[21]^ proposed a combined transfer of the anterior latissimus dorsi and teres major. Additionally, Baek *et al*^[21]^ reported on pectoralis major transfer for addressing anterior recurrent dislocation following RTSA^[^19–21^]^.

As salvage techniques, Tashjian *et al*^[^22^]^ described a high-strength suture cerclage between the proximal humerus and the scapula as an effective method to restore stability in recurrently unstable RTSA. Budge *et al*^[23]^ demonstrated that using an Achilles allograft for capsular reconstruction in cases of recalcitrant RTSA instability can yield a stable, functional shoulder for patients willing to accept a limited range of motion and function^[^22,23^]^.

Although this technique can be used as a salvage treatment, it may result in severe rigidity and a reduced range of motion. The authors believe that the hamstring autograft technique could provide better shoulder stability and improved functional outcomes.

We describe a different soft tissue reconstruction technique with a hamstring autograft for the management of instability in RTSA. Potential disadvantages of this approach include graft incorporation failure, donor site morbidity, and the risk of over-constraining the prosthesis; however, careful attention to graft placement may mitigate these risks.

## Conclusion

In this current case report, we detailed a persistent anterior dislocation after RTSA treated with an anterior sling hamstring autograft. In the setting of RTSA, subscapularis deficiency can occasionally present a challenging and notable complication. This anterior sling technique could be a potential treatment option when dealing with recurrent anterior dislocation after RTSA due to subscapularis deficiency.

## Data Availability

Not applicable.

## References

[1] Schairer WW Nwachukwu BU Lyman S. National utilization of reverse total shoulder arthroplasty in the United States. J Shoulder Elbow Surg 2015;24:91–97. 25440519 10.1016/j.jse.2014.08.026

[2] Abdelfattah A Otto RJ Simon P. Classification of instability after reverse shoulder arthroplasty guides surgical management and outcomes. J Shoulder Elbow Surg 2018;27:e107–18. 29175353 10.1016/j.jse.2017.09.031

[3] Chalmers PN Rahman Z Romeo AA. Early dislocation after reverse total shoulder arthroplasty. J Shoulder Elbow Surg 2014;23:737–44. 24188682 10.1016/j.jse.2013.08.015

[4] Kerwan A Al-Jabir A Mathew G. Revised Surgical CAse REport (SCARE) guideline: An update for the age of Artificial Intelligence. Prem J Sci 2025;10:100079.

[5] Boileau P Melis B Duperron D. Revision surgery of reverse shoulder arthroplasty. J Shoulder Elbow Surg 2013;22:1359–70. 23706884 10.1016/j.jse.2013.02.004

[6] Kohan EM Chalmers PN Salazar D. Dislocation following reverse total shoulder arthroplasty. J Shoulder Elbow Surg 2017;26:1238–45. 28162886 10.1016/j.jse.2016.12.073

[7] Testa EJ Glass E Ames A. Indication matters: effect of indication on clinical outcome following reverse total shoulder arthroplasty-a multicenter study. J Shoulder Elbow Surg 2024;33:1235–42. 37944747 10.1016/j.jse.2023.09.033

[8] Ascione F Schiavone Panni A Braile A. Problems, complications, and reinterventions in 4893 onlay humeral lateralized reverse shoulder arthroplasties: a systematic review (part I-complications). J Orthop Traumatol 2021;22:27. 34236540 10.1186/s10195-021-00592-w PMC8266956

[9] Wierks C Skolasky RL Ji JH. Reverse total shoulder replacement: Intraoperative and early postoperative complications. Clin Orthop Relat Res 2009;467:225–34. 18685908 10.1007/s11999-008-0406-1 PMC2600997

[10] Cheung EV Sarkissian EJ Sox-Harris A. Instability after reverse total shoulder arthroplasty. J Shoulder Elbow Surg 2018;27:1946–52. 29934280 10.1016/j.jse.2018.04.015

[11] Chae J Siljander M Wiater JM. Instability in reverse total shoulder arthroplasty. J Am Acad Orthop Surg 2018;26:587–96. 30074512 10.5435/JAAOS-D-16-00408

[12] Melbourne C Munassi SD Ayala G. Revision for instability following reverse total shoulder arthroplasty: outcomes and risk factors for failure. J Shoulder Elbow Surg 2023;32:S46–S52. 36822501 10.1016/j.jse.2023.01.023

[13] Markes AR Cheung E Ma CB. Failed reverse shoulder arthroplasty and recommendations for revision. Curr Ver Musculoskelet Med 2020;13:1–10. 10.1007/s12178-020-09602-6 PMC7083982 31956943

[14] Franceschetti E De Sanctis EG Ranieri R. The role of the subscapularis tendo in a lateralized reverse total shoulder arthroplasty: Repair versus nonrepair. Int Orthop 2019;43:2579–86. 30612172 10.1007/s00264-018-4275-2

[15] Friedman RJ Flurin PH Wright TW. Comparison of reverse total shoulder arthroplasty outcomes with and without subscapularis repair. J Shoulder Elbow Surg 2017;26:662–68. 28277259 10.1016/j.jse.2016.09.027

[16] De Fine M Sartori M Giavaresi G. The role of subscapularis repair following reverse shoulder arthroplasty: systematic review and meta-analysis. Arch Orthop Trauma Surg 2022;142:2147–56. 33635398 10.1007/s00402-020-03716-9

[17] Edwards TB Williams MD Labriola JE. Subscapularis insufficiency and the risk of shoulder dislocation after reverse shoulder arthroplasty. J Shoulder Elbow Surg 2009;18:892–96. 19282204 10.1016/j.jse.2008.12.013

[18] Collin P Rol M Muniandy M. Relationship between postoperative integrity of subscapularis tendon and functional outcome in reverse shoulder arthroplasty. J Shoulder Elbow Surg 2022;31:63–71. 34216783 10.1016/j.jse.2021.05.024

[19] Kany J. Tendon transfers in rotator-cuff surgery. Orthop Traumatol Surg Res 2020;106:S43–51. 31843509 10.1016/j.otsr.2019.05.023

[20] Baek CH Kim JG Baek GR. Restoration of active internal rotation following reverse shoulder arthroplasty: Anterior latissimus dorsi and teres major combined transfer. J Shoulder Elbow Surg 2022;31:1154–65. 34968688 10.1016/j.jse.2021.11.008

[21] Baek CH Kim BT Kim JG. Pectoralis Major Transfer For Anterior Recurrent Dislocation of Reverse Total Shoulder Arthroplasty: A Case Report. J Orthop Case Rep 2024;14:12–18. 10.13107/jocr.2024.v14.i06.4486 PMC11189068 38910979

[22] Tashjian RZ Broschinsky K Chalmers PN. Glenohumeral cerclage for salvage of recalcitrant instability after reverse total shoulder arthroplasty. J Shoulder Elbow Surg 2018;27:e259–63. 30016694 10.1016/j.jse.2018.05.001

[23] Budge MD. Instability of a reverse total shoulder arthroplasty treated with a novel allograft capsule reconstruction: a case report. JSES Rev Rep Tech 2022;3:107–10. 37588076 10.1016/j.xrrt.2022.09.001 PMC10426688

